# Diseases of the Thyroid Gland and Their Surgical Treatment

**Published:** 1901-12

**Authors:** 


**Diseases of the Thyroid Gland and their Surgical Treatment.** By JAMES BERRY, B.S. Lond., F.R.C.S. Pp. xvi., 367. London: J. & A. Churchill. 1901.

This book is based upon the essay to which the Jacksonian Prize of the Royal College of Surgeons for 1886 was awarded, and upon the Hunterian Lectures which the author delivered in 1891; and it is evidently the result of much patient work and considerable experience.

Although it mainly treats of the surgery of the thyroid gland, yet the book contains much interesting information as to the causation and distribution of endemic goitre, especially with reference to its geographical occurrence; and the author comes to the conclusion, which has been long held, that the true explanation lies in the geological structure of the ground and the influence of the water supply. He has found that endemic goitre is common among the Cotswold Hills, especially in the valleys. It is frequently met with in Stroud and its neighbourhood, also at Wotton-under-Edge and in many villages to the east of Cheltenham. It is also common at Clevedon and among the Mendip Hills. It does not seem to depend upon the hardness of the water, but goitre water always contains a large amount of total solid impurity. This theory as to the connection between drinking-water and endemic goitre has been often denied, and it is interesting to notice that so recent an investigator as Mr. Berry should return to the old theory. The most important part of the book is that which treats of the various operations for goitre. Enucleation is spoken of as the easiest and safest method in all cases of adenoma and in most cases of cystic disease. A word of warning is properly given as to the liability of recurrent hemorrhage after this operation. So many cases of this accident have occurred, that some surgeons are coming to the conclusion that extirpation is really the safer operation of the two; at all events, this liability should be borne in mind, and due care taken to avoid it as far as possible. Extirpation is fully described, and the various complications which may afterwards arise are plainly dealt with.

The author's operations for removal of goitre numbered 126, with only five deaths, and in his last 100 cases there was only one death. The book is the most complete on the subject of the surgery of the thyroid we have seen.

